# Cutaneous metastases as a primary manifestation of invasive ductal carcinoma of the breast: a case report

**DOI:** 10.1097/MS9.0000000000000835

**Published:** 2023-05-10

**Authors:** Nour Kara Tahhan, Anas Abou Azan, Isam Jomaa Al Ali, Jeer Abdul Aziz, Samer Sara

**Affiliations:** a Damascus University, Faculty of Medicine; b Department of surgery, Al-Mouwasat University Hospital, Damascus, Syria

**Keywords:** Breast cancer, case report, cutaneous metastases, invasive ductal carcinoma

## Abstract

Cutaneous metastases as the first sign of invasive ductal carcinoma are not common. The ambiguous presentation of asymptomatic lesions may result in various diagnoses including dermatologic causes. Early diagnosis is essential in such cases.

**Case presentation:**

A 43-year-old woman with no risk factors for developing breast cancer at a young age was diagnosed with invasive ductal carcinoma of the left breast after dermatologic complaints of diffuse lesions on the left-back and right subclavian region. The patient remained asymptomatic except for the recent cutaneous presentation, which did not arouse much suspicion.

**Conclusion:**

Cutaneous metastases of breast cancer remain uncommon, but at the same time represent a poor prognosis for the patient, and when they do occur, treatment options are limited. The delay in taking the proper diagnostic measures in such cases imposes a need to adopt a wider perspective when dealing with the possible occurrence of advanced disease. This also adds up to the importance of breast self-examination by women at a young age and full examination by physicians, especially when they encounter a misguiding presentation.

## Introduction

Highlights

- Keep in mind neoplasia in nonspecific cutaneous presentation that does not respond to treatment.
- Importance of breast self-examination even in patients with low risk of developing breast cancer.
- Running a full examination is essential in providing the best medical care available.

Breast cancer is the most diagnosed cancer and the second most common cause of cancer-related deaths in women^1^. The most common metastatic cancers are lung cancer in men and breast cancer in women^2^. Breast cancer is the most common solid malignancy that metastasizes to the skin^3^. The incidence of cutaneous metastasis from carcinomas ranges from 0.7 to 10.0%^4^. Breast cancer accounts for 30% of cases of cutaneous metastases. Cutaneous breast cancer metastases most commonly occur on the chest wall, abdomen, or, scalp^5^. Nodules are the most common form of cutaneous presentation^6^.

Previous studies found that 5–10% of patients with operable breast cancer develop chest wall recurrence within 10 years after mastectomy^7^.

Skin metastases usually develop near the primary tumour site by direct, lymphatic, or hematogenous invasion. However, cutaneous metastases rarely occur as the first manifestation of the cancer^5^.

Our patient was diagnosed with advanced breast cancer after cutaneous metastases vaguely appeared as the first evidence of cancer.

This raises the need to develop certain standards for the management of advanced cases such as this one, in which the available medical care is usually palliative.

This case report has been reported in line with the SCARE Criteria^8^.

## Presentation of case

### Patient information

A 43-year-old, healthy, single woman. First period at the age of 14 years. No previous marriages or pregnancies, no family history of breast cancer, and not enroled in a breast cancer screening program. The patient had regular menstruation and showed no symptoms of menopause or perimenopause.

The patient had a breast implant nine years ago for cosmetic reasons, which was removed after 1 year due to recurrent infections.

### Presentation

**Figure 1:**
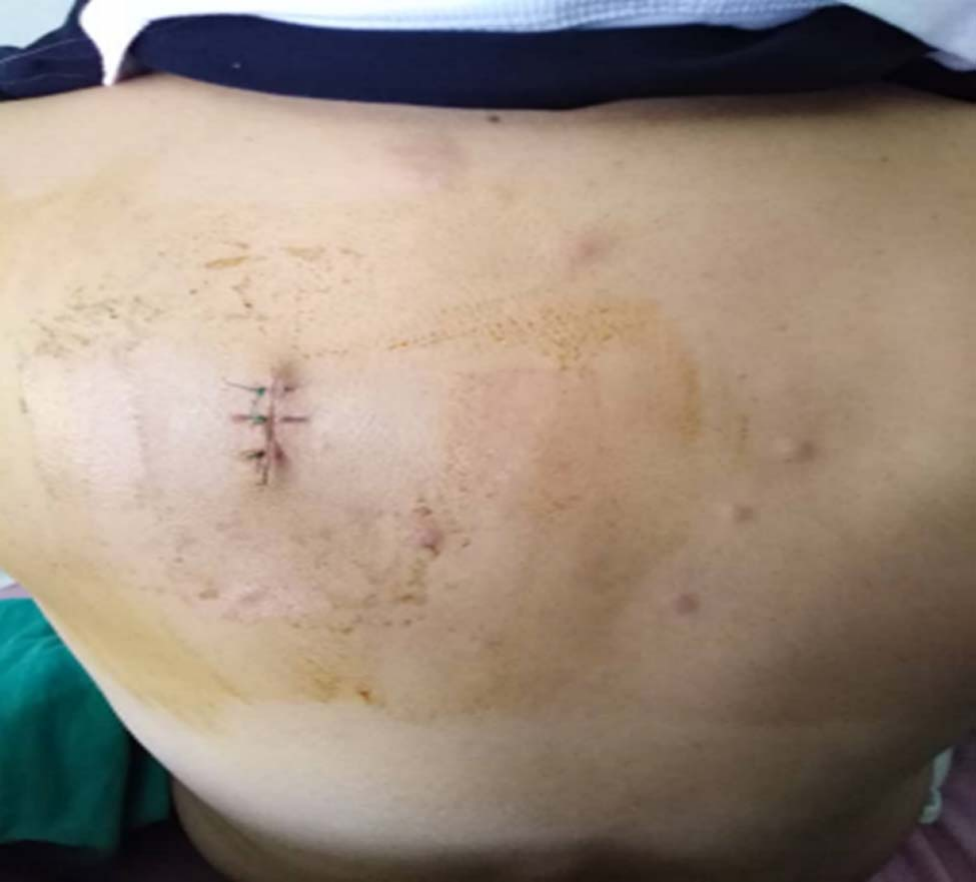
Cutaneous implantations in the upper back and right subclavian regions.

**Figure 2:**
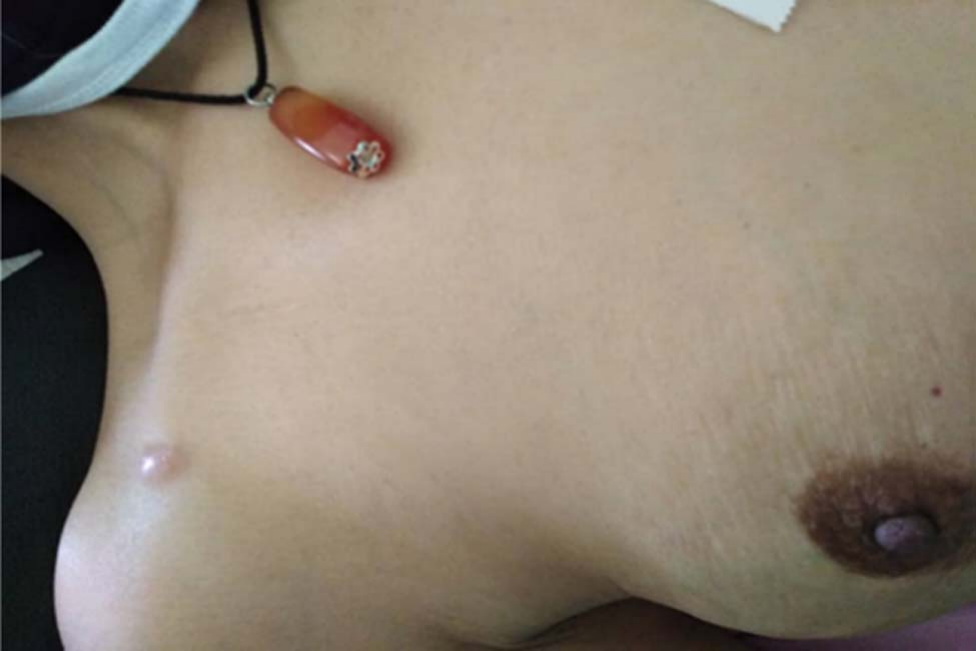
Cutaneous implantations in the upper back and right subclavian regions.

The patient complained of small, diffuse, pruritic lesions on her left upper back and right subclavian region that she noticed 1 month ago. (Figs. 1,2)

She had a dermatologist consult, who gave her a topical triamcinolone for 2 weeks, suspecting dermatitis that could be due to allergic reactions or eczema, but no improvement was noted.

After a complete breast examination, she was referred to our general surgery department for surgical evaluation of bilateral palped masses and retraction of the left nipple.

### Clinical findings

#### Physical examination

The patient underwent a complete examination and several investigations at our surgical department. On inspection, both breasts were small and had inferior bilateral surgical scars. The left nipple was retracted, while the right breast showed no significant changes. On palpation, a small irregular mass with indistinct margins was found in the lower outer quadrant of the left breast measuring ~2×3 cm, and a round mass measuring 3×3 cm was found in the upper outer quadrant of the right breast.

No palpable nodes were found in the axilla on either side. Diagnostic assessment/Investigations:

Ultrasound revealed round, hypoechoic, and diffuse lesions around the areola in the lower outer quadrant in both breasts, with the largest lesion measuring ~18.7×17×8 mm and showing evidence of interval growth. No posterior features, calcifications, vascularity, or cysts were found. Normal breast skin and mammary ducts were present.

Many reactive lymph nodes were found in the axilla, which had an echogenic fatty hilum and a thin cortex, with the largest lymph node measuring 15.8×7 mm and no suspicion of malignancy.

The breast findings were suggestive of fibrocystic changes, inflammatory alterations, abscesses, or fibrosis due to previous implantation.

On the other hand, cutaneous lesions suggested dermatitis, cysts, lipomas, metastases, or abscesses.

It was decided to perform a core biopsy of the suspicious mass in the left breast, which was classified as BI-RADS 4C, and an excisional biopsy on one of the skin lesions, in addition to essential lab tests.

The right mass was classified as BI-RADS 4A and a core biopsy was considered but the patient refused to undergo another breast biopsy.

The skin lesion in the right subclavian presented as pruritic skin infiltration. Therefore, no further investigations were recommended.

**Figure 3:**
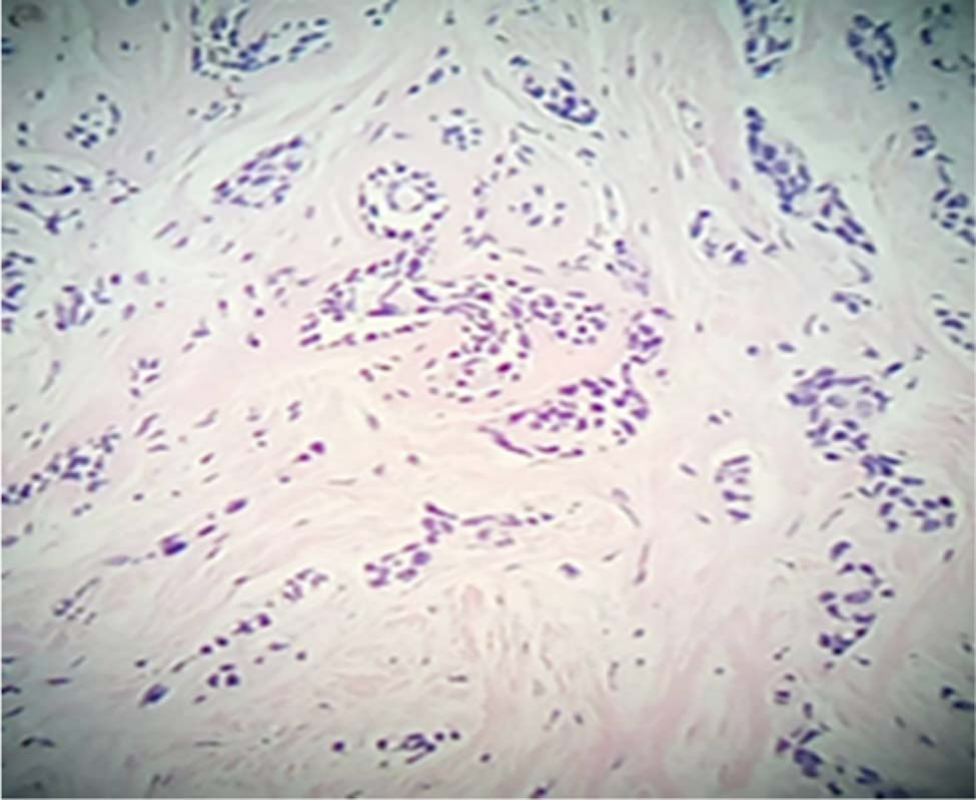
Left breast: tru-cut biopsy: invasive moderately differentiated ductal adenocarcinoma G2.

Laboratory results showed marked elevation of CA 15-3, lactate dehydrogenase, and Alkaline phosphatase. The tru-cut biopsy from the left breast revealed an invasive moderately differentiated ductal adenocarcinoma, G2. The tumour had a focal Ductal carcinoma in situ component and involved all tissue cores. (Fig. 3)

**Figure 4:**
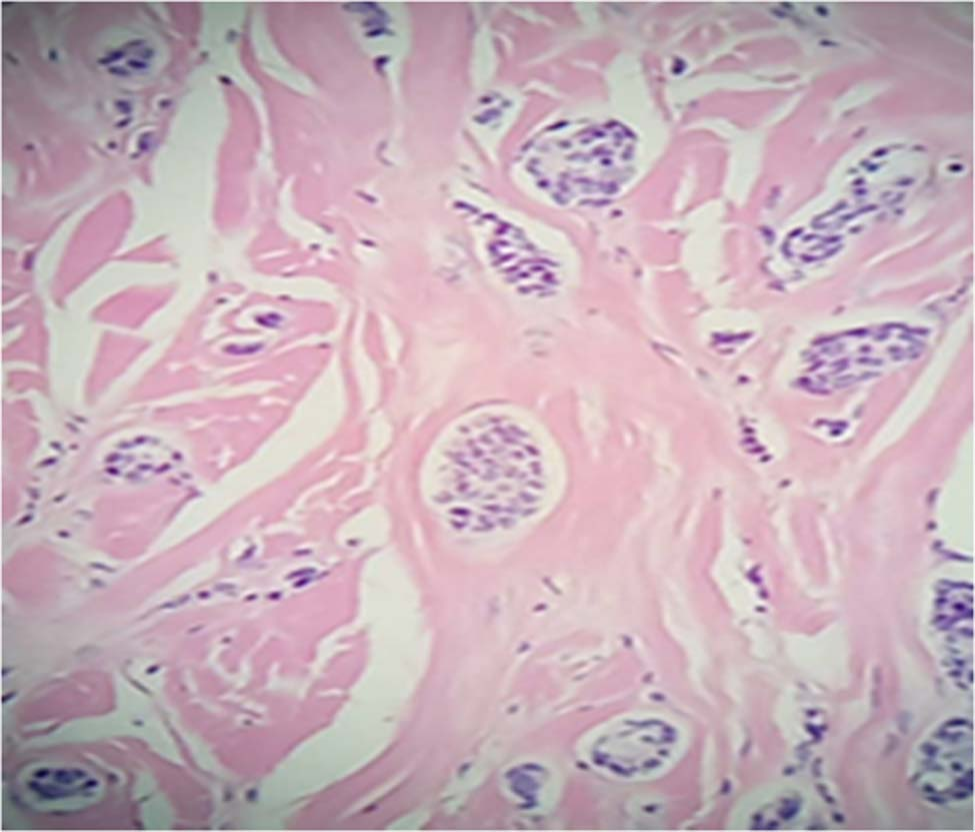
Back, skin lesion, resection: skin and subcutaneous adipose tissue involved with metastatic adenocarcinoma of breast origin.

Biopsy of the skin lesions revealed that the skin and subcutaneous adipose tissue were invaded by metastatic adenocarcinoma of the breast, focally extending to the surgical margins. Tests such as mammaglobin and GCDFP-15 were not available. (Fig. 4)

Tests on the breast tissue:

Hormone receptors were found: ER+, PR+.

HER2 status: negative.

Ki67 expression: not available.

The tumour was classified as stage IV breast cancer (T2, N0, M1).

Chest computed tomography (CT) revealed an undefined node in the lower outer quadrant of the right breast measuring of 2.5×3.5 cm, which required mammographic evaluation.

Several small bilateral nodes were found in the parietal and visceral areas, which could be suggestive of malignant metastases.

No pulmonary metastases or infusions were found.

Abdomen and pelvis CT showed two cysts in the left ovary, each 3 cm in size.

The liver, spleen, pancreas, kidneys, adrenals, and bladder were within normal ranges, and no hypertrophic lymph nodes or free fluid were found in the abdomen.

Images from CT also showed severe diffuse abnormal findings and osteolytic lesions suggestive of malignancy.

### Follow-up

The patient was referred to an oncology centre and treated with zoledronic acid, navelbine, and capecitabine. The patient completed 11 chemotherapies, but an increase in the number and size of bone lesions was discovered on CT , prompting Tc-MDP bone scintigraphy for further evaluation. The patient declined hormone therapy after being informed of the possible side effects. In addition, biomarker laboratory values were significantly elevated and cutaneous lesions showed no response to treatment. The patient declined IV chemotherapy and was referred to a palliative care unit. After 6 months, the patient developed multiple bone and visceral metastases with deterioration in her general condition, so she decided to discontinue treatment.

## Discussion

Cutaneous presentations as the first sign of breast cancer are still a rare condition, not often seen in practice. The fact that a nonspecific cutaneous lesion may hide an advanced-stage cancer that becomes multisystemic even before symptoms appear, must raise awareness of the importance of considering malignancies as silent causes of nonresponsive cutaneous lesions and including them in the differential diagnosis.

Cutaneous metastases of breast cancer usually involve the chest, abdomen, and scalp^5^, less commonly the back, upper arms, and lower abdomen; and rarely the buttocks, perianal region, lower extremities, and eyelids^9^. According to previous studies, cutaneous metastases were usually found solitary in patients older than 60 years. Breast cancer cutaneous metastases occurred more frequently as plaques and less frequently as nodules and were less often associated with multisystemic metastases^10^. The most common subtype metastasizing to the skin was invasive carcinoma of no special type^11^. In a previous study this was estimated to be 95.2%, followed by 2.4% of invasive lobular carcinoma^12^. Cutaneous metastases originating from the breast usually have a less poor prognosis than those originating from internal malignancies^5^. However, survival in these patients is short, with a median of 5 months^5,9^.

Our patient had multiple lesions presenting as nodules on the left-back, accounting for less than 10% of the distributions^13^, and had multiple metastases at diagnosis, which is a revelation in a case that deviates from the usual statistics.

Mammography screening is recommended in women older than 50 years, and since our patient had no family history of breast cancer, it is clear that our patient did not have important risk factors for screening for breast cancer at an earlier age.

Therefore, the patient did not participate in a screening program, so the breast mass could not be detected, and the dermatologist did not suspect malignant causes for the cutaneous lesions because the presentation was not specific for the lesion. The patient had no tumour-related symptoms, no clinical signs of inflammation, and the back is not a common region for metastases. This has led to ambiguous dermatological considerations of the disease, although cutaneous metastases from breast cancer are the most common metastases seen by dermatologists^14^.

The Syrian crisis may also have contributed to the delay in diagnosis due to limited self-awareness, capacity, and access to health services. In addition, breast self-examination was found to be low among Syrian women. There is still a need for more awareness in the Syrian society, which suffers from residual post-traumatic effects that include ignorance about one’s own health^15^.

Future physicians must think of neoplasia when a patient presents with a nonspecific clinical presentation that does not respond to treatment. It is also important to perform a complete examination and not limit it to the patient’s region of complaint, which is still probably waved in many institutions and clinics probably due to ethical and religious considerations of the patient, especially when it comes to the breast region. This sometimes leaves the physician with the choice of performing an incomplete clinical examination. In such cases, it is recommended that the patient be referred to a female physician to avoid a delayed or incorrect diagnosis if an incomplete examination is performed. This is important because in cases such as ours, the prognosis is poor, survival rates are low, and therapy is usually palliative^10^.

## Ethical approval

NA.

## Consent

Written informed consent was obtained from the patient’s parents for publication of this case report and any accompanying images. A copy of the written consent is available for review by the Editor-in-Chief of this journal.

## Source of funding

Not applicable.

## Author contribution

All authors contributed equally to this work.

## Conflicts of interest disclosure

No conflict of interest.

## Research registration unique identifying number (UIN)

This study is a case report, so we cannot make the registration it as a trial.

## Provenance and peer review

Not commissioned, externally peer-reviewed.
